# A novel *MAGED2* variant in a Chinese preterm newborn with transient antenatal Bartter’s syndrome with 4 years follow-up

**DOI:** 10.1186/s12882-021-02553-1

**Published:** 2021-12-11

**Authors:** Mingsheng Ma, Mengqi Zhang, Yu Zhou, Fengxia Yao, Min Wei, Zhenghong Li, Zhengqing Qiu

**Affiliations:** 1 Department of pediatrics, Peking Union Medical College Hospital, Chinese Academy of Medical Sciences & Peking Union Medical College, No 1, Shuaifuyuan, Dongcheng District, Beijing, 100730 China; 2 Laboratory of Clinical Genetics, Peking Union Medical College Hospital, Chinese Academy of Medical Sciences & Peking Union Medical College, Beijing, China

**Keywords:** *MAGED2* gene, Transient antenatal Bartter’s syndrome, Polyhydramnios, Case report

## Abstract

**Background:**

Transient antenatal Bartter’s syndrome caused by *MAGED2* mutation is a rare X-linked recessive renal tubular disorder. Cases reported are mostly infants, and the long-term prognosis of the disease is still under investigation.

**Case presentation:**

We encountered a preterm male infant with polyhydramnios, polyuria, salt loss, hypercalciuria, nephrocalcinosis and alkalosis. Antenatal Bartter’s syndrome was suspected, but these clinical symptoms surprisingly disappeared after about 2 months. This led to the clinical diagnosis of transient antenatal Bartter’s syndrome. Gene analysis in this patient disclosed a novel variant (c.1598C > T, p.Ala533Val) in exon 12 of *MAGED2* gene, and his mother was a heterozygous carrier. This patient was followed up in clinic for 4 years without recurrence of imbalance of potassium, sodium and chloride. His height and weight were in normal range, and all laboratory examinations and nephrotic ultrasound were also normal.

**Conclusions:**

We reported the first Chinese case of transient antenatal Bartter’s syndrome caused by *MAGED2* mutation. The 4-year follow-up of our case further demonstrates the benign prognosis of the disease and indicates that early recognition of this phenotype could avoid unnecessary treatments.

**Supplementary Information:**

The online version contains supplementary material available at 10.1186/s12882-021-02553-1.

## Background

Antenatal Bartter’s syndrome is an inherited disorder, characterized by polyuria, failure to thrive, hypokalemia, metabolic alkalosis, hyperreninemia and renal salt wasting which need lifelong fluid and electrolyte supplementation as well as nonsteroidal anti-inflammatory drugs. Some severe cases show mental retardation. The defective chloride transport in the loop of Henle leads to fetal polyuria resulting in severe hydramnios and premature delivery.

However, some case reports described male infants’ antenatal Bartter’s syndrome spontaneously resolved within several weeks after birth [1]. Laghmani et al. identified a genetic cause of this transient antenatal phenotype, *MAGED2* mutations by whole-exome sequencing of the suspected X chromosome [2]. The transient antenatal Bartter’s syndrome patients present with polyhydramnios, prematurity and renal salt tubular function, but these clinical manifestations disappeared completely in a few months after birth. Most patients reported were infants, and the long-term influence on kidney function was still unknown [3].

We identified a novel variant (c.1598C > T, p.Ala533Val) in *MAGED2* by next generation sequencing in a Chinese preterm neonate with transient antenatal Bartter’s syndrome phenotype. This patient was followed up in clinic for 4 years with regular test of kidney function and ultrasound.

## Case presentation

A previously healthy 31-year-old G1P0 woman presented with polyhydramnios at 22 weeks (amniotic fluid volume 11.5 cm, normal value < 8 cm) which led to a preterm delivery of a male infant at 29 weeks. The boy weighed 1480 g (P85) and was 38.5 cm (P50) long with head circumference 26.5 cm (P61). Polyuria (mean 5.8 ml/kg.h) was observed immediately after birth. Dehydration and rapid weight loss (decrease 25% at the fourth day after birth) followed. Persistent hyponatremia, hypokalemia, hypochloremia (Na^+^ 129 mmol/L, K^+^ 2.95 mmol/L, Cl^−^ 94 mmol/L) necessitated intravenous fluid infusion and supplemental electrolytes. Elevated levels of renin and angiotonin (PRA1 > 12 ng/ml/h, normal value range 0.05–0.79 ng/ml/h, AT-II1 > 800 pg/ml, normal value range 16.2-64 pg/ml) were noted. Severe hypercalciuria was identified by the ratio of Urine calcium:creatinine, which was 1147.8 mg/g Cr, and it further caused medullary nephrocalcinosis. Metabolic alkalosis progressively developed (maximal pH 7.521, BE 7.8 mmol/L, HCO3^−^ 31.1 mmol/L). The magnesium level in blood remained normal. Fortunately, he passed hearing screening by Otoacoustic emissions (OAE) and Auditory brainstem response (ABR). There was no positive family history for those symptoms.

Antenatal Bartter’s syndrome was suspected. He was treated with parenteral nutrition containing Na^+^ (maximum 4 mmol/kg, adjusting according to the concentration of Na^+^) and K^+^ (maximum 4.4 mmol/kg, adjusting according to the concentration of K^+^) until 21 days after birth. And then, oral 15% KCl solution (2 ml, three times per day, reduced gradually 3 weeks later), and spironolactone (2 mg/d) were given for about 1 month. All symptoms gradually relieved. Neonatal polyuria normalized within 4 days, and metabolic alkalosis disappeared after 1 month. After 2 months, serum electrolyte maintained normal without oral supplemental. Renin and angiotonin level also decreased to normal (PRA1 0.79 ng/ml/h, AT-II1 82.33 pg/ml) and kidney ultrasound showed improvement of medullary nephrocalcinosis. He weighed 3890 g (P40) and was 53 cm (P51) long with head circumference 35.5 cm (P53) at corrective gestational age of 42 weeks. Then, this patient was evaluated in clinic every 12 months. In the most recent follow-up when he was 4 years old, his height was 106.4 cm and weight was 15.8 kg. Complete blood test and kidney function tests were normal (Hgb 122 g/L, Na 138 mmol/L, K 4.0 mmol/L, Cl 103 mmol/L, creatine 31 μmol/L). kidney ultrasound revealed normal kidney size and structure.

**Fig. 1:**
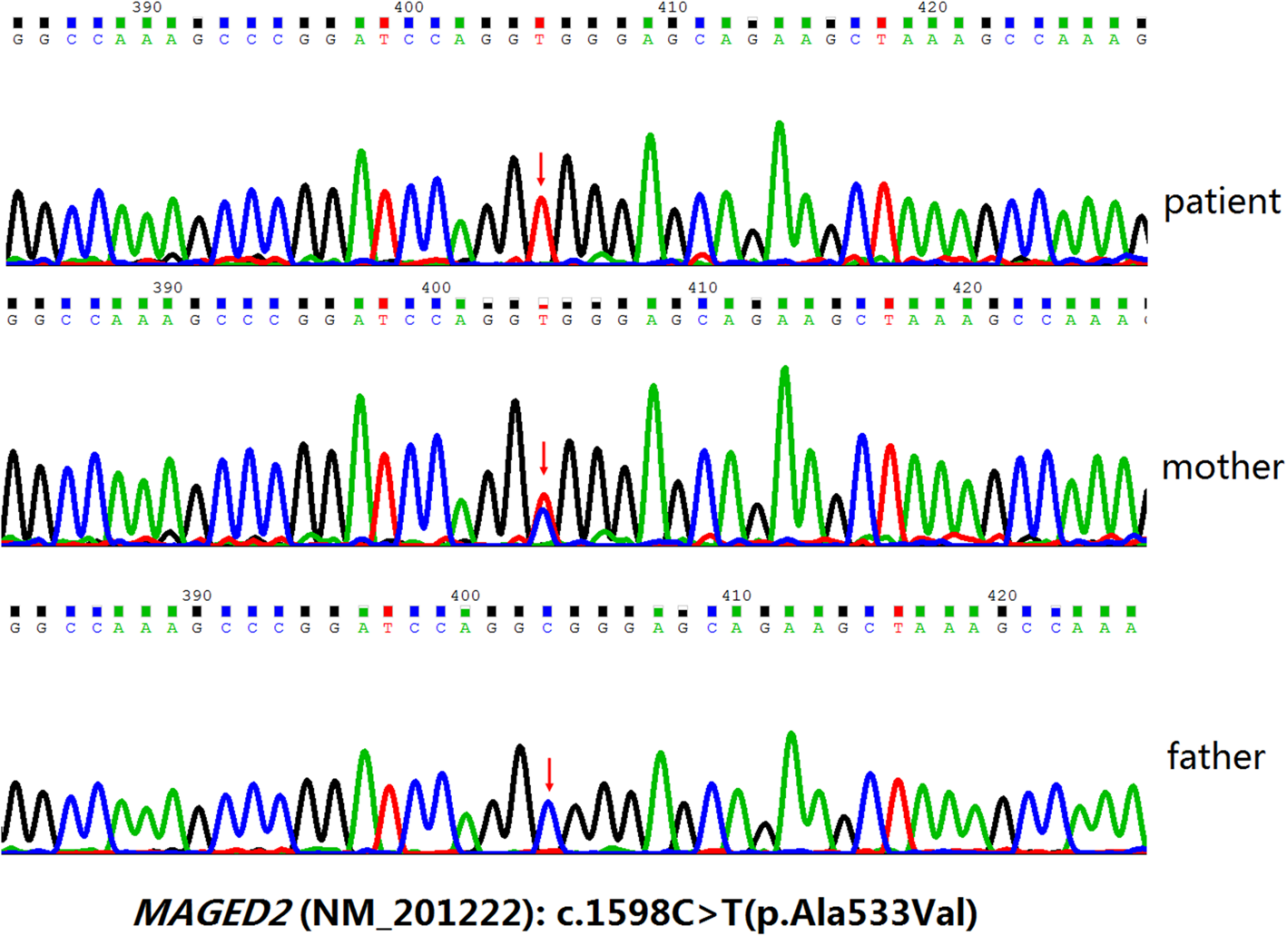
Sanger sequencing of MAGED2 gene in proband and family member. Blood samples were collected from the patient as well as his parents, and sequenced. Mutated *MAGED2* gene (c.1598C > T) was found in the patient 1 while his mother was a heterozygous carrier for that mutation

A congenital nephropathy gene panel including genes of classic and antenatal Bartter’s syndrome (270 genes listed in supplemental Table 1) was used to detect the pathogenic mutation. Genomic DNA was extracted from peripheral blood leukocytes of this patient and his parents according to the standard protocols for sequencing. On analysis of *MAGED2* gene, a variation(c.1598C > T, p.Ala533Val) detected by Next generation sequencing was confirmed by Sanger sequencing analyses (Fig. 1). This patient is hemizygous and his mother was a heterozygous carrier.

## Discussion and conclusions

In antenatal Bartter’s syndrome, five different genes are found to be responsible: *SLC12A19* (type I), *KCNJ1* (type II), *BSND* (type IV), *CLCNKA*, and *CLCNKB* (type IVb) [4]. Clinical manifestations include polyhydramnios, prematurity, polyuria, and renal salt wasting. Sensorineural deafness is also a manifestation of antenatal Bartter’s syndrome type IV and IVb. Treatment of lifelong electrolyte supplementation and nonsteroidal anti-inflammatory drugs is needed.

In May 2016, *MAGED2* was defined to be responsible for transient antenatal Bartter’s syndrome. *MAGED2* is located in Xp11.2 which is a hot spot for X-linked mental retardation (XLMR) and encodes melanoma-associated antigenD2 (MAGE-D2). MAGE-D2 is expressed universally in normal tissues and participates in cell cycle regulation. It protects cells from apoptosis [5]. This gene has been proven participating in development of breast cancer and melanoma and may be a promising biomarker for gastric cancer [6–8]. Laghmani et al. found MAGE-D2 also expresses in the distal tubules in kidneys and promotes NKCC2 and NCC expression through adenylate cyclase and cAMP signaling and a heat-shock protein [2]. Recent study reveals that MAGED2 mRNA and protein are significantly upregulated in tubular cells in human kidney injury, which indicates MAGED2 plays a role in tubular cell injury during acute kidney disease [9].

Most patients reported were infants, and the longest follow-up was 17 years with normal glomerular and tubular function [2]. All symptoms disappeared spontaneously during follow-up, but the mechanism of transient character of the disease is not clear. Two hypotheses were made by Laghmani et al. [2]. First, beyond a certain stage of renal development, MAGED2 is not needed for the expression of NKCC2 and NCC because of the increased sensitivity of adenylate cyclase activity to vasopressin. In addition, postnatally higher levels of oxygenation in the kidney may promote the synthesis of NKCC2 and NCC [10].

Various kinds of mutations associated with transient antenatal Bartter’s syndrome including nonsense, frameshift, splice-site mutations, missense and in-frame deletion, were identified and reported [2, 3, 11]. In this patient, a missense mutation was present. The variation, c.1598C > T locates in exon 12 of the *MAGED 2* gene. It predicts a change from an alanine codon to a valine codon. This variant was not found in the 1000 Genomes (http://1000genomes.org/) and Exac (http://exac.broadinstitute.org/), suggesting that it was not likely to be a Single Nucleotide Polymorphism (SNP). Polyphen-2 software was used to further evaluate the possible deleterious consequence of the novel MAGED2missense variant, and the p.Ala533Val substitution was predicted to be possibly damaging with a score of 0.780 which suggest the novel variant would substantially alter the function of protein. The results of multiple amino acid sequence alignment showed that the novel p.Ala533Val variant occurred at a highly evolutionarily conserved residue. Further study is needed to reveal the subsequent protein structure and functional change.

In this patient, diagnosis was made based on the transient clinical manifestations of antenatal Bartter’s syndrome. The differential diagnosis of this Bartter-like phenotype is other known types of antenatal Bartter’s syndrome (types I, II, IV, and IVb). According to the clinical manifestations of 13 patients with transient antenatal Bartter’s syndrome described by Laghmani et al., polyhydramnios appeared in second trimester of pregnancy, which was earlier than other known types of antenatal Bartter’s syndrome. All 13 patients with transient antenatal Bartter’s syndrome were preterm caused by polyhydramnios [2]. Polyuria lasted about 3 days to 6 weeks and ended at 30 to 33 weeks of gestational age. This phenotype is probably life threatening because of dehydration according to the reported cases. Bartter’s syndrome types I, II, IV, and IVb are associated with similar manifestations like polyhydramnios, prematurity. But polyuria, hypokalemia, and metabolic alkalosis persist and need lifelong treatment [12]. However, after 4-year follow-up in our hospital, our patient did not show any relapse of this diseases, suggesting a favorable prognosis of transient antenatal Bartter’s syndrome if the patient received carefully supporting treatment during the acute phase of this disease. Thus, we highlight the importance of early recognition of transient antenatal Bartter’s syndrome for saving lives as well as avoiding unnecessary and potentially harmful indomethacin treatment.

In conclusion, we report here the first Chinese case of transient antenatal Bartter’s syndrome and a novel *MAGED2* gene alteration. Gene analysis is recommended for preterm infant with prenatal severe polyhydramnios, postnatal polyuria and hypokalemia. Our 4 years follow-up of the patient further demonstrates a favorable prognosis of the disease and thus, early recognition of this transient disease helps to avoid unnecessary treatments.

## Supplementary Information

**Additional file 1.**

## Data Availability

A congenital nephropathy gene panel including genes of classic and antenatal Bartter’s syndrome (270 genes listed in supplemental Table 1) for next generation sequencing was used to detect the pathogenic mutation. The primers and conditions for thermal cycling, and the datasets used during the study are available from the corresponding author on reasonable request.

## Abbreviations

ABR: Auditory brainstem response
OAE: Otoacoustic emissions
MAGE-D2: melanoma-associated antigen D2
XLMR: X-linked mental retardation
