# Supplementary material for: A novel MAGED2 variant in a Chinese preterm newborn with transient antenatal Bartter’s syndrome with 4 years follow-up
Source: BMC Nephrol. 2021 Dec 11;22:408. doi: 10.1186/s12882-021-02553-1 (PMC8665482; doi:10.1186/s12882-021-02553-1)
Supplement: Supplementary file 1 — Additional file 1. [file 12882_2021_2553_MOESM1_ESM.docx]

Supplemental Table 1 Detailed 270 genes of congenital nephropathy gene panel

| *ACE* | *BUB1B* | *CTNS* | *HPD* | *NEK1* | *RPGRIP1L* | *TSC1* |
| --- | --- | --- | --- | --- | --- | --- |
| *ACTN4* | *C1QA* | *CUL3* | *HPRT1* | *NEK8* | *SALL1* | *TSC2* |
| *ADAMTS13* | *C1QB* | *CYP24A1* | *HSD17B4* | *NIPBL* | *SARS2* | *TTC21B* |
| *ADCK4* | *C1QC* | *CYP27B1* | *IFT122* | *NPHP1* | *SCARB2* | *TTC8* |
| *AGT* | *C3* | *CYP2R1* | *IFT140* | *NPHP3* | *SCNN1A* | *UMOD* |
| *AGTR1* | *C4A* | *DGKE* | *IFT172* | *NPHP4* | *SCNN1B* | *UPB1* |
| *AGXT* | *C5orf42* | *DMP1* | *IFT27* | *NPHS1* | *SCNN1G* | *VDR* |
| *AHI1* | *CA2* | *DNASE1* | *IFT43* | *NPHS2* | *SDCCAG8* | *VHL* |
| *ALDOB* | *CASR* | *DYNC2H1* | *IKBKAP* | *NR3C2* | *SLC12A1* | *VIPAS39* |
| *ALG1* | *CC2D2A* | *EGF* | *INF2* | *OCRL* | *SLC12A3* | *VPS33B* |
| *ANKS6* | *CCND1* | *EHHADH* | *INPP5E* | *OFD1* | *SLC17A5* | *WDPCP* |
| *ANLN* | *CD151* | *ENPP1* | *INVS* | *PAX2* | *SLC22A12* | *WDR19* |
| *APOA1* | *CD2AP* | *ESCO2* | *IQCB1* | *PDSS2* | *SLC2A2* | *WDR34* |
| *APOE* | *CD46* | *EVC* | *ITGA8* | *PEX1* | *SLC2A9* | *WDR35* |
| *APOL1* | *CEP120* | *EVC2* | *ITGB4* | *PEX10* | *SLC34A1* | *WDR60* |
| *APRT* | *CEP164* | *EXOC8* | *JAG1* | *PEX12* | *SLC34A3* | *WDR73* |
| *AQP2* | *CEP290* | *EYA1* | *KAT6B* | *PEX13* | *SLC37A4* | *WFS1* |
| *ARHGAP24* | *CEP41* | *FAH* | *KCNJ1* | *PEX14* | *SLC3A1* | *WNK1* |
| *ARHGDIA* | *CEP83* | *FAM20C* | *KIF14* | *PEX16* | *SLC4A1* | *WNK4* |
| *ARL13B* | *CFB* | *FCGR2B* | *KIF7* | *PEX19* | *SLC4A4* | *WT1* |
| *ARL6* | *CFH* | *FGA* | *KL* | *PEX2* | *SLC5A2* | *XDH* |
| *ATP6V0A4* | *CFHR1* | *FGF20* | *KLHL3* | *PEX26* | *SLC7A7* | *XPNPEP3* |
| *ATP6V1B1* | *CFHR3* | *FGF23* | *LAMB2* | *PEX3* | *SLC7A9* | *ZMPSTE24* |
| *ATP7B* | *CFI* | *FLCN* | *LCAT* | *PEX5* | *SLC9A3R1* | *ZNF423* |
| *AVPR2* | *CISD2* | *FLNB* | *LMBRD1* | *PEX6* | *SMARCAL1* |  |
| *B9D1* | *CLCN5* | *FN1* | *LMX1B* | *PEX7* | *SOX17* |  |
| *B9D2* | *CLCNKA* | *FXYD2* | *LPIN1* | *PHEX* | *STAR* |  |
| *BANK1* | *CLCNKB* | *G6PC* | *LYZ* | *PIGA* | *STRA6* |  |
| *BBIP1* | *CLDN16* | *GALE* | *LZTFL1* | *PIGT* | *TCTN1* |  |
| *BBS1* | *CLDN19* | *GALK1* | *MAGED2* | *PKD1* | *TCTN2* |  |
| *BBS10* | *CNNM2* | *GALNT3* | *MKKS* | *PKD2* | *TCTN3* |  |
| *BBS12* | *COL4A3* | *GALT* | *MKS1* | *PKHD1* | *THBD* |  |
| *BBS2* | *COL4A4* | *GATA3* | *MMAA* | *PLCE1* | *TMEM138* |  |
| *BBS4* | *COL4A5* | *GLA* | *MMAB* | *PMM2* | *TMEM216* |  |
| *BBS5* | *COL4A6* | *GLB1* | *MMACHC* | *PNP* | *TMEM231* |  |
| *BBS7* | *COQ2* | *GLIS2* | *MMADHC* | *PRPS1* | *TMEM237* |  |
| *BBS9* | *COQ6* | *GPC3* | *MUC1* | *PTPN22* | *TMEM67* |  |
| *BCOR* | *CRB2* | *GRHPR* | *MUT* | *PTPRO* | *TNXB* |  |
| *BCS1L* | *CSPP1* | *HNF1B* | *MYH9* | *REN* | *TREX1* |  |
| *BMP4* | *CTH* | *HNF4A* | *MYO1E* | *RET* | *TRIM32* |  |
| *BSND* | *CTLA4* | *HOGA1* | *NAA10* | *ROBO2* | *TRPC6* |  |
